# Women’s Subjective Experiences of Living with Vulvodynia: A Systematic Review and Meta-Ethnography

**DOI:** 10.1007/s10508-017-1026-1

**Published:** 2017-09-13

**Authors:** Rebekah Shallcross, Joanne M. Dickson, David Nunns, Catharine Mackenzie, Gundi Kiemle

**Affiliations:** 1 Doctorate of Clinical Psychology Programme, The University of Liverpool, Liverpool, UK; 2 Centre for Academic Primary Care, School of Social and Community Medicine, The University of Bristol, Room 2.03 Canynge Hall, 39 Whatley Road, Bristol, BS8 2PS UK; 3 Department of Psychological Sciences, University of Liverpool, Liverpool, UK; 4 Department of Psychology, Edith Cowan University, Joondalup, Australia; 5 Department of Gynaecological Oncology, Nottingham University Hospitals, Nottingham, UK; 6 The Vulval Pain Society, Nottingham, UK

**Keywords:** Vulvodynia, Vulval/Vulvar pain, Meta-ethnography

## Abstract

Vulvodynia, the experience of an idiopathic pain in the form of burning, soreness, or throbbing in the vulval area, affects around 4–16% of the population. The current review used systematic search strategies and meta-ethnography as a means of identifying, analyzing, and synthesizing the existing literature pertaining to women’s subjective experiences of living with vulvodynia. Four key concepts were identified: (1) Social Constructions: Sex, Women, and Femininity: Women experienced negative consequences of social narratives around womanhood, sexuality, and femininity, including the prioritization of penetrative sex, the belief that it is the role of women to provide sex for men, and media portrayals of sex as easy and natural. (2) Seeking Help: Women experienced the healthcare system as dismissive, sometimes being prescribed treatments that exacerbated the experience of pain. (3) Psychological and Relational Impact of Vulvodynia: Women experienced feeling shame and guilt, which in turn led to the experience of psychological distress, low mood, anxiety, and low self-esteem. Moreover, women reported feeling silenced which in turn affected their heterosexual relationships and their peer relationships by feeling social isolated. (4) A Way Forward: Women found changing narratives, as well as group and individual multidisciplinary approaches, helpful in managing vulvodynia. The findings of the review conclude that interventions at the individual level, as well as interventions aimed at equipping women to challenge social narratives, may be helpful for the psychological well-being of women with vulvodynia.

## Introduction

Vulval pain is the experience of pain in the form of burning, soreness, or throbbing in the vulval area (Nunns & Murphy, 2012). It has been classified by the International Society for the Study of Vulvovaginal Diseases into two categories, namely pain caused by specific, identifiable, and underlying disorders and idiopathic pain in the absence of identifiable underlying disease (Harlow & Stewart, 2003). The term vulvodynia refers to the latter classification and is the focus of this review.

Quantitative research demonstrates that vulvodynia is very common, affecting 25% of all women at some point in their lifetime and around 8% of women at any one time (Reed et al., 2012), with other estimates of prevalence varying from 4 to 16% (Eppsteiner, Boardman, & Stockdale, 2014). It affects women of any age (Nunns & Murphy, 2012), with rate of the first onset greatest before the age of 25 (Harlow et al., 2014). Prevalence is similar for African-American women and white women; however, Hispanic women are around 1.4 times more likely to experience vulvodynia (Harlow et al., 2014). The reasons for this are unknown. Despite the relatively high prevalence, little is known about the underlying etiology, with several causative factors likely, including (but not limited to) embryology, neuropathic pain, and infection, which may all contribute to the experience of pain differently for different women (for a review, see Eppsteiner et al., 2014). Furthermore, quantitative research examining the possible causative experience of a history of sexual abuse produces inconsistent findings, making firm conclusions difficult to draw (Reed et al., 2000).

Obtaining a diagnosis of vulvodynia is often time-consuming and difficult, most likely due to diagnostic complexity plus a lack of awareness among healthcare professionals (Toeima & Nieto, 2011). Treatment has tended to have a biomedical focus based upon expert opinion, clinical experience, and observational studies, as very few randomized control trials exist (Ayling & Ussher, 2008). Treatment includes skin care guidance, topical and oral medications, surgery, physiotherapy, and psychotherapy, with a multidisciplinary approach often needed for successful treatment (Eppsteiner et al., 2014). Quantitative research into the physical experience of vulvodynia demonstrates numerous impacts, including experiences of discomfort and/or severe pain, impinging on a variety of activities such as using tampons, engaging in penetrative vaginal sex, wearing tight clothing, practicing sports, or even everyday activities such as sitting, walking, or sleeping (Ponte, Klemperer, Sahay, & Chren, 2009; Reed, 2006). Furthermore, quantitative research into the experience of pain demonstrates how vulvodynia also impacts psychosocial functioning, with women who experience vulvodynia suffering from increased rates of anxiety, depression, sexual dissatisfaction, and reduced self-esteem (Gates & Galask, 2001).

While clearly contributing to our understanding of vulvodynia, the research into women’s experiences of vulvodynia outlined above is quantitative in nature, with some studies suffering methodological limitations such as lack of control groups, or vague inclusion/exclusion criteria (Marriott & Thompson, 2008). Moreover, quantitative methodology, by its nature, requires converting social phenomena to numerical values in order to carry out statistical analysis. Qualitative methodology, on the other hand, aims to explore, describe, and interpret the personal and social experiences of participants (Smith, 2007), offering broader insights into how women may experience vulvodynia. The current review therefore aims to summarize the qualitative literature pertaining to women’s experiences of vulvodynia.

However, before reviewing this qualitative literature, it is helpful to provide a brief overview of the broader feminist literature pertaining to women’s sexuality and the medicalization of women’s bodies, in order to provide a contextual backdrop to the experiences that women with vulvodynia likely face, given the nature of their pain and the nature of the medical environment in which they find themselves.

There are several key areas which would be useful to visit here, including: the coital imperative, phallocentrism, and patriarchal ideologies (Du Plessis, 2015; Exner, Dworkin, Hoffman, & Ehrhardt, 2003; McPhillips, Braun, & Gavey, 2001), the hydraulic male sex drive assumption (explained below) (Gavey, McPhillips, & Doherty, 2001; Vitellone, 2000), the negating medical profession, and the medicalization of women’s bodies (Marken, 1996; Tiefer, 2001).

The “coital imperative” is the notion that “real” sex equals penetration of the vagina by the penis (coitus) and it places this particular sexual act as central to “normal” heterosex (Jackson, 1984; McPhillips et al., 2001). “Phallocentrism” and “patriarchal ideologies” in this area have been defined in feminist sexology research as, respectively, viewing penile erections as the essence of male sexuality and satisfaction, and the expectation of female submission to provide pleasure and meet the sexual as well as the emotional needs of men (Du Plessis, 2015). Feminist sexology research provides examples of how each of these factors can impact upon women’s experience of sexuality, often in a limiting way. For example, while reviewing sexology literature into HIV and AIDS prevention, Exner et al. (2003) highlighted several detrimental narratives surrounding sex, including men as sexual initiators and orchestrators (Byers, 1996) and a universal sexual double standard that gives men greater sexual freedom and rights of sexual determination than women (Blanc, 2001), both of which contribute to prohibiting women from owning their own sexuality and asserting their own needs or desires (Segal, 1994). Moreover, feminist sexology research has begun to examine the way in which the media have replaced traditional regulators of sexual practice (historically religious leaders) as the authority on normal sexual practice (Du Plessis, 2015). Specifically, Du Plessis, using discourse analysis, concluded in an examination of media outlets that the media are still underpinned by patriarchal notions of male sexuality which limit both male and female sexuality to predefined gender relation such as the penis as an icon of sexual pleasure for both men and women; “real” sex equals penile–vaginal penetration and anything other than this is “foreplay” and therefore of secondary importance; men have a “need” for sex that is biological and innate and women are obliged to satisfy it, which all restrict women’s sexuality to a framework that is inflexible and limited in possibilities.

The hegemonic heterosexual male sex drive is presented as an innate need for penetrative sex, something to be satiated by women and prioritized over their own pleasure, as well as being “non-negotiable, spontaneous and uninterruptible” (Vitellone, 2000, p. 156). Research into the promotion of safer sex has explored how the condom is seen as feminizing as its use by men represents a demonstration of a degree of control over sexual behavior that is in conflict with the idea of the male sex drive as an innate, uninterruptible, and powerful hydraulic force (Vitellone, 2000). Similarly, campaigns such as the “If it’s not on, it’s not on” slogan portray an underlying assumption that women must take responsibility for condom use (i.e., that “it’s on”), reconstituting the ideology of male (hetero)sexuality as an uninterruptible, hydraulic instinct which must be satisfied (Gavey et al., 2001; Vitellone, 2000). As such, narratives of masculine identity that go unchallenged prevent both condom use and non-penetrative sexual activities, leaving little room for sexual satisfaction to be gained from anything other than coitus for both men and women limiting men and women’s experiences when it comes to sex and pleasure.

Feminist sexology researchers argue that much research into sexuality is rooted in male perspectives and understandings (Amaro, Rai, & Reed, 2001; Exner et al., 2003), therefore, often negating women’s experiences and their needs. This arguably has translated into clinical practice, with the literature suggesting that doctors may perceive female patients as “inherently dependent” and “lacking in common sense,” a view that rationalizes “paternalistic attitudes and advice” (Gannon, 1998, p. 295). Similar research highlights a male bias in medicine, whereby the unchanging male body that maintains a state of equilibrium and stability is seen as “normal,” while the constantly changing female body (through menstruation, pregnancy and menopause) is seen as pathological (Marken, 1996). Prior to the medicalization of women’s bodies, women’s complaints were often explained away as being “all in their heads,” insanity or hysteria (Marken, 1996; Tasca, Rapetti, Carta, & Fadda, 2012). The themes of the medical profession viewing women through a paternalistic lens and as pathological by nature, while ignoring social structures that negatively impact upon women, should be borne in mind when considering the experiences of women with vulvodynia.

In sum, previous feminist literature has already identified several key areas that may be pertinent to women’s experiences of vulvodynia including the coital imperative, the male hydraulic sex drive, and phallocentrism. Quantitative research to date has focused on the prevalence of vulvodynia, with estimates varying from four to sixteen percent of women, and there is a lack of understanding of the etiology of the disease, with several causative factors likely (Eppsteiner et al., 2014). Further, obtaining a diagnosis of vulvodynia is time-consuming and difficult, partly due to a lack of awareness in healthcare professionals (Toeima & Nieto, 2011), and while a variety of treatment options have been researched, evidence regarding effectiveness above and beyond placebo is nominal, with individualized, multidisciplinary approaches likely to be needed, along with future research into how these can be most effectively developed (Eppsteiner et al., 2014).

Therefore, given the complexity and seeming necessity for an individualized assessment and management of vulvodynia, along with the need for further research into which multidisciplinary approaches are likely to be of benefit to women, the current review aims to develop a broader understanding of women’s experiences of vulvodynia by reviewing and analyzing the existing, yet sparse, qualitative literature regarding women’s experiences of vulvodynia, thus developing an understanding of women’s personal and social experiences of living with vulvodynia, using a qualitative method of synthesis: meta-ethnography.

## Method

The current review aimed to explore, analyze, and summarize the experiences of women in relation to any aspect of living with vulvodynia, in order to further our understanding by answering the question: What are women’s subjective experiences of living with vulvodynia?

### Search Methods and Inclusion Criteria

A systematic search strategy and meta-ethnography were employed as a means of identifying and synthesizing all relevant literature.

**Fig. 1:**
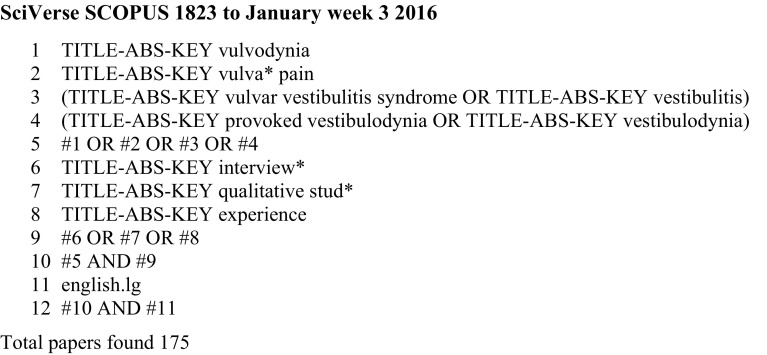
Example search terms

**Fig. 2:**
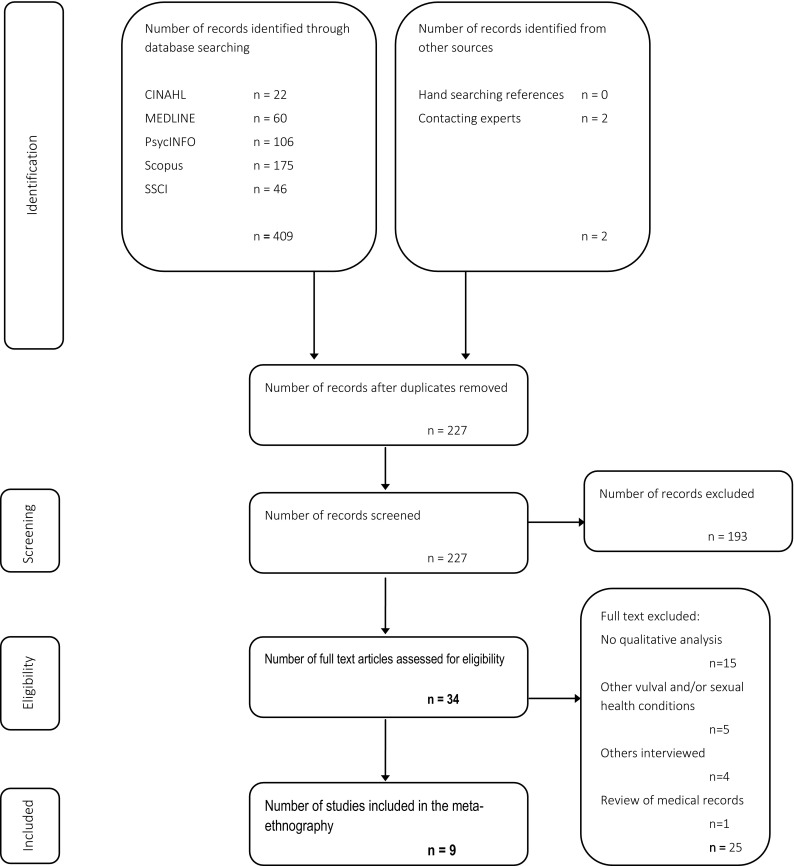
Identification of papers flow diagram [Adapted From: Moher, Liberati, Tetzlaff, Altman and The PRISMA Group (2009).]

The following online databases (and interfaces) were searched on January 21, 2016, using developed search strategies synonymous with “vulva* pain” OR “vulvodynia” OR “vestibulodynia” OR “vestibulitis” AND “interview*” OR “qualitative stud*” OR “experience”: MEDLINE (Ovid); CINAHL plus (EBSCO*host*); Scopus (SciVerse); PsycINFO (EBSCO*host*); and Social Sciences Citation Index (SSCI; Thomson Reuters). Figure 1 outlines an example search. Experts in the field were contacted to further identify missing papers. References were hand searched, and literature referenced on the Vulval Pain Society website was examined for possible inclusion. The inclusion criteria for papers were: utilization of unstructured or semi-structured qualitative interviews and qualitative analysis methods; exploration of the views of women with a diagnosis of idiopathic vulval pain (vulvodynia, vestibulodynia, vulvar/vulval vestibulitis); and obtainment of full text published in English, in peer-reviewed journals. Figure 2 outlines the stages of the search process.

### Search Outcome

Table 1 details the included studies. A total of 227 papers were identified, and all abstracts and titles screened for relevance, with those not meeting the inclusion criteria excluded. Following the initial screening, 34 papers remained and the full manuscripts of these were obtained. The study inclusion/exclusion criteria were applied to each remaining paper by author RS, and also independently rated between a further three researchers RD, JE, and CT (see Acknowledgments). Agreement was met between researchers for the nine papers included in this literature review, from which 185 women were interviewed.

**Table 1:** Included studies and study characteristics

Study characteristics	Ayling and Ussher	Brotto et al.	Buchan et al. and Munday et al.	Kaler	Marriott and Thompson	Sadownik et al. (a and b)	Johnson et al.
Year	2008	2013	2007	2006	2008	2012	2015
Title	“If sex hurts, am I still a woman?”: The subjective experience of vulvodynia in heterosexual women	Impact of an integrated mindfulness and cognitive behavioral treatment for provoked vestibulodynia (IMPROVED): A qualitative study	A qualitative study of women with vulvodynia I. the journey into treatment & II. Response to a multidisciplinary approach to management	Unreal women: Sex, gender, identity, and the lived experience of vulvar pain	Managing threats to femininity: Personal and interpersonal experience of living with vulval pain	Provoked vestibulodynia: a qualitative exploration of women’s experiences & Provoked vestibulodynia: women’s experiences of participating in a multidisciplinary vulvodynia program	“You have to go through and have your children”: reproductive experiences among women with vulvodynia
Sample size	*n* = 7	*n* = 14	*n* = 29	*n* = 20 face-to-face interviews; *n* = 70 open-ended web-based interactions	*n* = 8	*n* = 19	*n* = 18
Average age	27 (18–41)	40 (21–68)	36 (22–58)	Late teens to mid-50s; with the majority mid-20s to mid-30s	27 (18–41)	31 (20–54)	32 (26–40)
Diagnoses	Vulvodynia	Provoked Vestibulodynia	All had provoked, localized vulvodynia and some also had unprovoked generalized vulvodynia	Primary and secondary vulvodynia	Vulvar vestibulitis	Provoked vestibulodynia	*n* = 17 had been formally diagnosed with vulvodynia (any type). *n* = 1 had symptoms consistent with vulvodynia
Length of symptoms (range)	*n*3 = 2–5 years; *n*4 = 5–10 years; all had a diagnosis <6 months previously	*n* = 6 lifelong; *n* = 8 acquired (2–26 years)	–	–	Mean 3 years (8 months–14 years)	Mean 65.24 months (6–240 months)	–
Relationship status and sexuality	*n*6 = currently partnered (4 of which cohabiting); *n*1 = recently been in heterosexual relationship	*n* = 9 partnered (mean duration 7.2 years).	–	63% of web participants were either married or living with a long-term male partner 2 interview and 2 web participants identified themselves as bisexual and 1 web participant identified herself as a lesbian	*n* = 7 heterosexual relationship; *n* = 1 single	*n* = 10 married; *n* = 5 common law; *n* = 1 divorced; *n* = 3 single	All participants were currently or previously in heterosexual relationships; 17 of the 18 (94%) currently had a male partner
Socioeconomic details	*n*5 = tertiary educated	All post-secondary education with two having graduate degrees	Educated	A variety of socioeconomic background with 37% of web participants with a post-secondary education	*n* = 4 employed; *n* = 1 full-time mother; *n* = 3 students	*n* = 18 post-secondary education; *n* = 1 high school education	*n* = 9 master’s or doctoral degree. Nearly half had some college education whether or not they obtained a degree, and 1 did not answer the question
Ethnicity/nationality	*n*7 = Anglo Saxon-Australian	*n* = 9 European Ancestry; *n* = 2 East Asian	White British or White European	White American	*n* = 8 Caucasian, British	*n* = 11 Euro-Canadian; *n* = 6 South Asian; *n* = 2 Middle Eastern	USA cohort. All respondents for which this information was provided (*n* = 16; 89% of the total sample), were white
Data collection (sample and setting, interview/focus group)	Vulvar pain clinic in Sydney, Australia; Semi-structured face-to-face interviews and demographic questionnaire	Large metropolitan west coast city, Canada; Face-to-face or telephone semi-structured interview	Vulvar pain service, Watford, UK; Semi-structured in-depth interview; Home/clinic/university	Newsletter of national vulvodynia association, related websites, and medical professional; interviews or web-based interactions	NHS specialist vulval pain clinic, Britain; Semi-structured interview in clinic or home	Multidisciplinary vulvodynia program in Vancouver, Canada; semi-structured interview in clinic or home	Recruited from a 2010–2012 national online, prospective cohort study that investigated vulvar pain across pregnancy and into postpartum
Inclusion/exclusion criteria	No gynecological surgery; unrelated to vulvodynia; no diagnosed chronic pain unrelated to vulvodynia	Completed the IMPROVED study Diagnosis of provoked vestibulodynia	–	Women with primary or secondary vulvodynia	Pre-menopausal Diagnosis of vulval vestibulitis	Pre-menopausal/reproductive age; no differential diagnosis; able to speak sufficient English	English speaking; aged 18–45; diagnosis of, or symptoms consistent with vulvodynia prior to pregnancy; within the first 15 weeks of gestation or given birth in the last 6–12 months
Theoretical perspective/epistemological standpoint	Critical realist epistemology	–	–	Feminist efforts to theorize gender	Assumption that it is possible to access an individual’s cognitive inner world	–	–
Qualitative method of analysis	Thematic analysis/material discursive approach	Content analysis	Thematic coding	Discursive analysis	Interpretative phenomenological analysis	Thematic	Sequence of events framework to develop themes
Aims and hypotheses	To investigate the “psycholic symptomology” of women with vulvodynia from a discursive perspective 1. what subject positions do heterosexual women with vulvodynia take up in relation to their sexuality when coital sex is limited or painful 2. in what ways do these subject positions impact upon women’s negotiation of material discursive aspects of vulvodynia with a heterosexual relationship	To explore the qualitative experiences of women taking part in group treatment program that integrates mindfulness principles along with cognitive behavioral therapy	To evaluate women’s experiences of accessing help and treatment for vulvodynia (Buchan et al.) To evaluate the response of a group of women with vulvodynia who were participating in an integrative multidisciplinary management programme comprising of medical evaluation and treatment, psychotherapy, physiotherapy, and dietary advice (Munday et al.)	To take up the lives of women with persistent vulvar pain for what they can reveal about the enmeshment of gender (hetero)sexuality and bodily practices	Explore the meaning women make of their experiences of having and being treated for vulval pain Explore the impact of vulval pain on their relationships	To explore the quality of our patients’ lives and the quality of their interactions with the healthcare system prior to enrollment in the multidisciplinary vulvodynia programme (Sadownik et al. a) To explore the experiences of women who participated in the multidisciplinary vulvodynia programme in order to identify the perceived benefits of this programme (Sadownik et al. b)	To explore how vulvodynia affects the following topics before, during and after pregnancy: 1. Clinician relationships and roles 2. Partner relationships and roles; 3. Control over pain and treatment regimen decision-making; 4. Concerns and expectations regarding pregnancy; and 5. Motherhood

### Quality Appraisal and Critique of Papers

No paper was excluded on the basis of the quality assessment, so as not to limit the potential for new insights to be found. However, the Critical Appraisal Skills Programme (CASP, 2014) tool for qualitative research was utilized in order to structure critique of the papers included and to inform on the strengths and limitations of any such insights. Table 2 provides an overview of the quality of each paper included in the study. The nine papers identified relate to seven studies, with papers by Buchan, Munday, Ravenhill, Wiggs, and Brooks (2007) and Munday, Buchan, Ravenhill, Wiggs, and Brooks (2007) reporting outcomes of one study, and papers by Sadownik, Seal, and Brotto (2012a, b) reporting outcomes of another study. Only three studies provided information pertaining to the epistemological standpoint of the authors (Ayling & Ussher, 2008; Kaler, 2006; Marriott & Thompson, 2008); the remaining authors failed to adequately discuss this. All the studies constitute valuable research and contribute to the existing knowledge.

**Table 2:** Quality appraisal-based on the CASP tool

	Ayling and Ussher (2008)	Brotto et al. (2013)	Buchan et al. (2007), Munday et al. (2007)	Kaler (2006)	Marriott and Thompson (2008)	Sadownik et al. (2012a, b)	Johnson et al. (2015)
*Screening questions*							
Does the paper report on findings from qualitative research and did that work involve both qualitative methods of data collection and data analysis?	✓	✓	✓	✓	✓	✓	✓
Is the research relevant to the synthesis topic?	✓	✓	✓	✓	✓	✓	✓
*CASP questions*							
Was there a clear statement of the aims of the research?	✓	✘	✓	✓	✓	✓	✓
Is a qualitative methodology appropriate?	✓	✓	✓	✓	✓	✓	✓
Was the research design appropriate to address the aims of the research?	✓	✓	✗	✓	✓	✓	✓
Was the recruitment strategy appropriate to the aims of the research?	✓	✓	✓	✓	✓	✓	✓
Was the data collected in a way that addressed the research issue?	✓	✓	✓	✓	✓	✓	✓
Has the relationship between researcher and participants been adequately considered?	✓	✘	✘	✘	✘	✘	✘
Have ethical issues been taken into consideration?	✓	✓	✓	✓	✓	✓	✓
Was the data analysis sufficiently rigorous?	✓	✓	✘	✘	✓	✓	✓
Is there a clear statement of findings?	✓	✓	✘	✓	✓	✓	✓
How valuable is the research?	Valuable	Valuable	Valuable	Valuable	Valuable	Valuable	Valuable
*Modified question*							
Do the researchers consider and adequately discuss their epistemological stand point?	✓	✘	✘	✓	✓	✘	✘

### Data Abstraction and Synthesis

Data were abstracted and synthesized using meta-ethnography (Noblit & Hare, 1988), guided by a previously published worked example (Britten et al., 2002). Meta-ethnography aims to produce a synthesis that demonstrates how original studies included in a given review are conceptually organized in relation to one another. In essence, the key concepts (stand-out ideas, themes, and interpretations within the original study) are treated as data. The key concepts are analyzed to determine whether they are reciprocal across studies (using reciprocal analysis: do they have similar concepts that support one another?) or refutational (using refutational analysis: do they differ from and refute each other?). A table is constructed to facilitate this process (see Table 3). Finally, from this process, a “line of argument” is developed which represents novel third-order interpretations, thus, further contributing to the existing literature above and beyond the summation of the concepts from the original papers.

**Table 3:** Table to aid analysis

	Ayling and Ussher	Brotto et al.	Buchan et al. and Munday et al.	Kaler	Marriott and Thompson	Sadowink et al. (a and b)	Johnson et al.
*Key concept 1: societal construction: sex, women and femininity*							
The coital imperative	Belief that real sex = coitus Coitus in a sexual relationship is “normal” Continue to have [real] sex despite pain	–	Penetrative sex still attempted; Stopping any physical contact for fear that it may lead to [real] sex	Belief that real sex = intercourse; Belief that biologically we are supposed to be having sex	Continuing to have painful sex; Avoidance of any intimacy which may lead to sex; Centrality of sex within relationships	Avoidance of physical intimacy	Continuing to have painful sex in order to conceive
The one thing that men really want	Idea that my partner is more understanding than other men would be; Men have a biological need for coitus which is prioritized	–	Most women described their partner as supportive (but still worried they were unable to give them [real] sex)	A good man is hard to find; Sex as a commodity that men want (and women with vulvodynia cannot offer)	Prioritization of male partners; Perceived male biological need for [real] sex	–	–
Not [a] real [woman] & loss	(loss) of identity for attracting men; Putting up with/not hurting a man’s feelings being a good (real) woman	–	Loss of sexual desire Social isolation (loss of) self-esteem (loss of) confidence	Idea that women’s authenticity comes from their relation to men; Not a “real” woman; Not a “real” marriage—absence of a “real” first time	Loss of femininity; Femininity analogous with sex	–	“not [being] a real woman”; not being capable of hav[ing] sex or do[ing] womanly sexual activities Fertility difficulties, conception, and delivery all different with vulvodynia
Media portrayal of women	Media portray women as wanting of sex Media portray sex as easy Young people should be having lots of sex	–	Media representations of “normal” sex lives reminding women of what they cannot achieve	No anger at heteronormative ideologies Women as sex objects Media portrayal of femininity/heterosexuality/normality	Perception that for others sex is fun and easy (in the media)	–	–
*Key concept 2: seeking help*							
Iatrogenesis	–	No improvement or worsening of symptoms	Recurrent treatment which impacted on psychological well-being	–	Repeated thrush treatment	Repeated visits with same treatment	Improvements in symptoms as women moved away from the medical treatments and healthcare systems
Experience of the medical model	–	Alienation of single women	Attitude of physicians left women feeling emotionally disturbed/self-questioning Relief of diagnosis; Despair at no cure	Anger at medical profession Told by physicians: need to relax neurotic stop fretting	Judgement from the medical profession; Prioritization of a medical diagnosis	The neurotic woman/not a valid complaint Diagnosis only did a little	Needing to be their own expert and advocate; Made to feel “crazy”; lack of health professional knowledge; fragmented system
The mind/body split	–	A concern that if vulvodynia is psychological then it’s not “real”	–	Feeling of the body “knowing” and trying to communicate; A desire to detach self from the body–	Medical = external & removable; Psychological = something “wrong” with a core aspect of myself “all in my head”	Made to feel that the pain was “all in my head” and therefore “not real”	Made to feel “it’s all in your head”
*Key concept 3: the psychological and relational impact of vulvodynia*							
Psychological distress, shame, and guilt	Shame, despite partners understanding	Guilt at not practising suggested techniques	Shame/embarrassment	Feeling of “otherness”	Fear of rejection Humiliation and shame	Psychological distress	Exhaustion, disheartened, anxiety, embarrassment
Silencing of women	Non-communication was barrier for exploring other ways of having sex	–	Inability to talk to others about vulvar pain Need to hide sexual problems Unspoken collusion (to avoid sex) became the norm	Difficulty bonding with “real” women (difficulty communicating)	–	–	Suggestion that the process could be improved by better patient/doctor communication suggesting that communication was not encouraged
*Key concept 4: a way forward*							
A practical difference	–	Normalization and empowerment from groups	Empowerment; Regaining control; self-efficacy over improvement from groups	–	–	Empowerment; increased knowledge via information and skill learning (CBT);—validation/support	Acceptance and normalization of pain in childbirth
A difference in narrative	Focusing on other “womanly” qualities (caring/nurturing etc.); Feminist/egalitarian discourse allowing the questioning the position of “inadequate woman”	–	–	Adopting other “traditional” woman qualities, i.e., caring and a good listener	–	–	–

## Results

The demographic details of participants are outlined in Table 1. It is of note that all studies had a majority Caucasian and heterosexual sample; were conducted in “Western countries” (Canada, U.S., Australia, and UK), which could be argued to have relatively similar cultural, economic and political influences; and showed a trend for women taking part to be “educated.”

The methods of analysis and aims of each study differed. Ayling and Ussher (2008) and Kaler (2006) both used discursive analysis, aiming to investigate the subject positions that women take up in relation to their sexuality when coitus is painful, and to understand more about the enmeshment of gender and (hetero)sexual practices, respectively. Similarly, Marriott and Thompson (2008) used interpretative phenomenological analysis to explore the meaning women make of their experiences with vulvodynia. In contrast to this, Johnson, Harwood, and Nguyen (2015) used framework analysis to produce themes, while Brotto, Basson, Carlson, and Zhu (2013), Buchan et al. (2007), Munday et al. (2007), and Sadownik et al. (2012a, b) all used thematic/content analysis in order to evaluate the experiences of women before and after participating in various multidisciplinary group interventions. It may therefore be expected that the findings of the papers may differ due to the differing, albeit qualitative, methodologies.

Four key concepts were identified: (1) Societal Constructions: Sex, Women, and Femininity, (2) Seeking Help, (3) Psychological and Relational Impact of Vulvodynia and (4) A Way Forward. The studies all related to one another through contribution to the following key concepts.

### Key Concept 1: Societal Constructions: Sex, Women, and Femininity

This key concept refers to experiences that women described in their interviews, which authors of the original papers identified and labeled as constructions that were unhelpful to women experiencing vulvodynia. Within the concept, four sub-concepts were identified:

#### Sub-concept 1: The Coital Imperative

The women discussed all acts of physical intimacy in relation to the “coital imperative” (McPhillips et al., 2001; Potts, 2002), which posits that “real sex” equals coitus: penetration of the vagina by the penis (Ayling & Ussher, 2008, p. 298).

Six out of seven studies described a theme surrounding the notion of the “coital imperative” (Ayling & Ussher, 2008; Buchan et al., 2007; Johnson et al., 2015; Kaler, 2006; Marriott & Thompson, 2008; Sadownik et al., 2012b). Women with vulvodynia across the studies held the view that sex was only “real,” “proper,” or “normal” if it involved coitus (Ayling & Ussher, 2008; Kaler, 2006; Marriott & Thompson, 2008). These beliefs impacted upon women who “grieved over their inability to engage in sexual intercourse” (Sadownik et al., 2012b, p. 25) or “believed they were expected to accept it” (Buchan et al., 2007, p. 18). As a result of these beliefs, and in some cases the desire to conceive a child, women continued to engage in painful penetrative sex: “You have to go through it and have your children that you wanted to have anyway” (Johnson et al., 2015, p. 7). Kaler (2006) and Ayling and Ussher (2008) suggested that a desire to be normal and the normative role that intercourse plays is “a critical factor in hetero-sexual women’s experiences of vulvodynia” (Ayling & Ussher, 2008, p. 299), with the “coital imperative” excluding “any positive positions for heterosexual women who cannot, or who choose not to, participate in coitus” (Ayling & Ussher, 2008, p. 301).

#### Sub-concept 2: The One Thing That Men Really Want

The subject position of “inadequate sexual partner” was associated with adherence to the “male sex drive” discourse, which defines man’s “need” for coitus as a biological drive which his female partner must accommodate (Holloway, 1989; Nicolson & Burr, 2003; Potts, 2002) (Ayling & Ussher, 2008, p. 298).

This sub-concept was identified in four of the seven studies (Ayling & Ussher, 2008; Buchan et al., 2007; Kaler, 2006; Marriott & Thompson, 2008). It refers to a belief held by participants that men have a “need” for sex and “would be upset by lack of sex” (Marriott & Thompson, 2008, p. 249). Furthermore, women believed it was a woman’s “duty” to satisfy that need (Ayling & Ussher, 2008; Marriott & Thompson, 2008). As such, women “privileged” their partner’s “need” for penetrative sex over their own “need for, or right to, pain-free sex” (Ayling & Ussher, 2008, p. 299).

Moreover, many women “positioned their current partner as more supportive and understanding than they imagined other men might be” (Ayling & Ussher, 2008, p. 299; Buchan et al., 2007; Kaler, 2006; Marriott & Thompson, 2008). Despite this, participants continued to hold the contradictory belief that “understanding men” were “rare and difficult to find” (Kaler, 2006, p. 68). The authors used this contradiction to confront women’s perceptions of their partners’ “need” for coitus, instead suggesting that women’s perception that they are unable to provide “the one thing that men really want” could be challenged (Kaler, 2006, p. 60).

#### Sub-concept 3: Not [a] Real [Woman] and Loss

In response to the question “Does vulvodynia affect your sense of self as a woman?” women expressed a sense of themselves as “degendered” and “defeminized” by vulvodynia (Kaler, 2006, p. 60).

Five out of seven studies (Ayling & Ussher, 2008; Buchan et al., 2007; Johnson et al., 2015; Kaler, 2006; Marriott & Thompson, 2008) reported themes relating to a sense of “loss of femininity” (Marriott & Thompson, 2008, p. 248), not being capable of “doing womanly sexual activities” (Johnson et al., 2015, p. 9.), or an “inability to claim womanhood as an identity” (Kaler, 2006, p. 62), which was specifically linked to women’s inability to engage in intercourse and to the ability to “attract and keep a man” (Ayling & Ussher, 2008, p. 299). As such, authors reported that women’s sense of femininity was defined by whether men would view them as desirable, with some women viewing themselves as “something no man would desire if he knew what he was getting himself into” (Kaler, 2006, p. 64). Kaler (2006) suggested that heterosexuality can be seen as a “set of market relations and transactions in which men “contracted” for sex with women” (p. 71). Therefore, women may perceive (consciously or unconsciously) a loss of “currency” (sex), due to difficulties in “performing” coitus. Women therefore worried about “false advertising” regarding sexual intercourse (Kaler, 2006, p. 64), risking “punitive consequences, such as the derogatory labels of ‘frigid’ or ‘prick tease’ or a diagnosis of ‘sexual dysfunction” (Marriott & Thompson, 2008, p. 301), perhaps further contributing to women’s feelings of being “fake” or “pseudo-women” and confirming themselves as “unreal women” (Kaler, 2006, p. 63).

#### Sub-concept 4: Media Portrayal of Women

This was exacerbated by media representations, especially in women’s magazines, of what constituted “normal” sex lives. It reminded them of what they could not achieve and perpetuated the feeling of failure (Buchan et al., 2007, p. 17).

Five of the seven studies referred to themes relating to perceived societal or cultural norms regarding sex, often perpetuated by media representations of how sex and relationships “should” be (Ayling & Ussher, 2008; Buchan et al., 2007; Johnson et al., 2015; Kaler, 2006; Marriott & Thompson, 2008). These societal norms and perceptions cultivated negative self-evaluations in women with vulvodynia, leading to a sense of alienation in comparison to “other ‘healthy’ heterosexual women” for whom sex (or indeed conception and birth of children; Johnson et al., 2015) was perceived to be “easy and natural,” further leaving women with a feeling that “I’m having all these problems” and “the rest of the world isn’t” (Marriott & Thompson, 2008, p. 249). In particular, the portrayal of young women in the media as “skillful,” “eager” (Ayling & Ussher, 2008, p. 229) and “even more sexually active because of their youth” (Marriott & Thompson, 2008, p. 249) was highlighted as impacting negatively upon study participants, leaving them feeling “inexperienced,” “immature,” and “constrained by the material limits of their vulval pain” (Ayling & Ussher, 2008, p. 229).

### Key Concept 2: Seeking Help

Five studies described themes relating to the key concept of Seeking Help. This key concept is comprised of three sub-concepts; (1) iatrogenesis, (2) negative experiences of the medical model and (3) the mind/body split, all of which relate to women’s experiences of seeking help for their vulvodynia.

#### Sub-concept 1: Iatrogenesis

“Iatrogenesis” or an “iatrogenic effect” refers to an “illness caused by medical examination or treatment” (oxforddictionaries.com). Four studies (and five papers) presented findings relating to this sub-concept (Brotto et al., 2013; Buchan et al., 2007; Johnson et al., 2015; Marriott & Thompson, 2008; Munday et al., 2007). Three studies described how women reported being repeatedly prescribed thrush treatment in the absence of candida (yeast) infections (Brotto et al., 2013; Buchan et al., 2007; Marriott & Thompson, 2008), while Johnson et al. described how participants described their pain as becoming more under self-control as “they moved away from medical treatments and healthcare systems” (p. 9). At best, women described how repeated thrush treatment had no effect on their experience of pain (Marriott & Thompson, 2008) and, at worst, women described a worsening of symptoms (Brotto et al., 2013; Buchan et al., 2007).

Women described side effects of certain medications (e.g,. amitriptyline), such as weight gain, which led to the experience of iatrogenic difficulties affecting their self-esteem and sexuality (Munday et al., 2007), and attributed delays in treatment/referrals as having a direct causative detrimental effect on their mental health (Buchan et al., 2007).

#### Sub-concept 2: Negative Experiences of the Medical Model

Like most women in the study, Sue directed her strongest emotions against medical professionals and institutions which women believed had belittled and misdiagnosed vulvodynia, rather than at the more nebulous and hard-to-pin-down targets of hegemonic norms of gender and sexuality (Kaler, 2006, p. 69).

Six out of seven studies outlined themes relating the experience of the medical model. Women reported seeing multiple healthcare professionals who had very little knowledge about vulvodynia (Buchan et al., 2007; Johnson et al., 2015; Marriott & Thompson, 2008; Sadownik et al., 2012a, b). Specifically, surrounding pregnancy, women felt that knowledge about the impact of vulvodynia was lacking, with advice often being contradictory to women’s experiences (Johnson et al., 2015). Moreover, women felt they were mistreated by healthcare professionals because of their gender, often experiencing the suggestion that they were “crazy,” “neurotic,” “frigid,” needed to “relax,” or that sex was just painful for women (Johnson et al., 2015; Kaler, 2006; Marriott & Thompson, 2008; Sadownik et al., 2012b), which left women feeling “emotionally disturbed and self-questioning” (Buchan et al., 2007, p. 17), and experiencing feelings of “anger,” “shame,” and “stigma” (Marriott & Thompson, 2008, pp. 254–255).

#### Sub-concept 3: The Mind/Body Split

Some women felt that there was more hope of overcoming the pain if it were physical in origin, but that it was almost hopeless if it were psychological. It seemed that a medical condition could be externalized and hopefully removed, but that a psychological aspect of the pain indicated something wrong with them “internally,” in the core aspects of themselves (Marriott & Thompson, 2008, p. 252).

Five studies outlined themes that pertained to women’s distress at the implication that the pain was “all in their head” (Brotto et al., 2013; Johnson et al., 2015; Kaler, 2006; Marriott & Thompson, 2008; Sadownik et al., 2012b). Kaler (2006) theorizes about this, suggesting that women “expressed a yearning” for the “mind/body split,” and that women “wanted a way to disconnect the troubled body from the real, essential self, an assurance that our bodies, to twist the well-known phrase, are not ourselves” (Kaler, 2006, p. 67).

### Key Concept 3: The Psychological and Relational Impact of Vulvodynia

Women described both interpersonal effects of vulvodynia (relationships with others) and intrapersonal effects of vulvodynia (relationship with themselves). This key concept was found within all seven studies and is comprised of the sub-concepts: (1) psychological distress, shame, and guilt and (2) silencing women.

#### Sub-concept 1: Psychological Distress, Shame, and Guilt

Participants reported negative changes in their mood, including frustration, anxiety, stress, and depression (Sadownik et al., 2012b, p. 25).

Psychological distress was a prevalent theme across all seven studies. Buchan et al. (2007), Johnson et al. (2015), Sadownik et al. (2012a), and Brotto et al. (2013) all report psychological difficulties in the women they interviewed, including low self-esteem, fear, anxiety, frustration, an altered self-image, inadequacy, guilt, shame, and “depression that varied from low mood to clinical depression requiring antidepressant medication” (Buchan et al., 2007, p. 17).

However, while also noting the experiences of these psychological difficulties in their studies, Ayling and Ussher (2008), Kaler (2006) and Marriott and Thompson (2008) all move beyond the identification of psychological difficulties, suggesting that these difficulties are not solely brought about by the experience of pain or the inability to have sex per se, but rather as a result of social narratives and discourses (outlined in key concept 1) that contribute largely to the negative psychological experiences of women with vulvodynia. For example, Marriott and Thompson (2008) postulated that women with vulvodynia experience shame due to the social contextualization of their evaluation of themselves, as they perceive others to see them (i.e., abnormal or “other”), which in turn leads to the experience of low mood and anxiety (Gilbert, 2006). Similarly, Ayling and Ussher (2008) noted that women reported experiences of “shame” in spite of support from partners. They suggest that this contradiction occurs due to significant discourses around coitus establishing heterosexual relationships as normal, regardless of the material context of an intimate partner who is supportive and non-pressurizing. Moreover, they found that women who did position themselves as an “inadequate woman/sexual partner” experienced “guilt, shame, and a decreased desire for sexual contact.” In contrast, one woman, through use of egalitarian discourse, was able to challenge these unhelpful discourses and as such reported being “confident” and “happy” (Ayling & Ussher, 2008). Finally, Kaler (2006) specifically addressed the experience of guilt that women with vulvodynia experience, suggesting that the objectification of women as sexual objects leads them to feel a sense of inauthenticity or guilt: “from the outside world, they might be mistaken for a proper sexual object, being attractive and desirable, but on the inside they were “bad” or “spoiled” (Kaler, 2006, p. 64).

Moreover, as discussed in Key Concept 2, the authors of the papers report that iatrogenic medication, negative interactions with the healthcare system, and the notion of the mind/body split also exacerbated the experience of psychological distress in these women with vulvodynia. As such, it is likely that the experience of socially contextualized shame, based upon social narratives of what constitutes “normal” gender and sexuality, as well as interactions with the healthcare system, may play a key role in moderating the psychological difficulties experienced by women with vulvodynia.

#### Sub-concept 2: Silencing of Women

Shame is highly disempowering and can result in self-silencing and isolation (Seu, 1995). Combined with the taboo associated with female genitalia and the discussion of sexual practices, this can act to constrain women from seeking professional help for their symptoms, or from communicating honestly with their partners and experimenting with alternative forms of intimacy (Ayling & Ussher, 2008, p. 301).

Three studies discussed the difficulty faced by women in communicating openly about their experiences of vulvodynia (Ayling & Ussher, 2008; Buchan et al., 2007; Kaler, 2006). Women seemed to understand that they needed to remain silent about their vulvodynia, a necessity that Ayling and Ussher (2008) attribute to the taboo surrounding sex, particularly women and sex, while Johnson et al. (2015, p. 7) allude to embarrassment as being a factor in women remaining silent. Indeed, in one study, some women had not talked to anyone about their pain (Buchan et al., 2007), highlighting the pressure experienced by women to remain silent about their experiences. In relation to this, in one study, Buchan et al. (2007) described how the lack of communication would lead to a situation where the “women were in fear of experiencing pain and so avoided sex, and partners were fearful of causing pain, and therefore an unspoken collusion to avoid sex became the norm” (p. 17). Similarly, Johnson et al. (2015, p. 7) reported that several participants suggested that “open communication between patient and provider nurtured by mutual recognition of the emotional aspects of pain” would improve their experiences with the healthcare profession.

The self-silencing that women engaged in rendered them socially isolated and thus unsupported, and as such, vulvodynia modified women’s relationships with other women, as well as their relationships with men. Kaler (2006) observed that women with vulvodynia often reported finding themselves excluded or separated from communities of women, whereby “if a woman had no heterosex to talk about, she could find herself an outsider in communities of women structured by heteronormative discourses” (Kaler, 2006, p. 65).

The action, or rather inaction, of women remaining silent about their vulvodynia, has serious psychological consequences, with Buchan et al. (2007) concluding that women’s need to hide their vulvodynia from peers and social networks “exacerbated their feelings of abnormality, social isolation, and difference; that further eroded their social identity, self-esteem, and confidence” (Buchan et al., 2007, p. 17). Moreover, previous literature suggesting that self-silencing is linked to increased rates of depression (Jack, 1991) is used as evidence by Ayling and Ussher (2008) to explain, to some degree, why women with vulvodynia experience psychological difficulties including, “in extreme cases, suicidal ideation” (Ayling & Ussher, 2008, p. 301).

### Key Concept 4: A Way Forward

The final key concept, A Way Forward, was referenced in four studies (Ayling & Ussher, 2008; Brotto et al., 2013; Buchan et al., 2007; Munday et al., 2007; Sadownik et al., 2012a), and is comprised of the sub-concepts (1) a practical difference and (2) a different narrative.

#### Sub-concept 1: A Practical Difference

Women reported that there were some practical changes that allowed them to “move forward” in relation to their vulvodynia, which focused on two main areas: (1) increasing communication with partners and (2) empowerment and control.

Women who attended Brotto et al.’s (2013) integrated mindfulness and cognitive behavioral group reported that the information provided at these sessions about vulvodynia enabled women to “open up a dialogue” with partners through sharing the information and being helped to define and talk about the vulvodynia. The women reported that this impacted upon the quality of their relationships by increasing understanding and “relationship cohesiveness” (Brotto et al., 2013, p. 10). In support of this, addressing feelings of shame in therapy and providing women with information was also found to facilitate communication with partners in other studies (Buchan et al., 2007; Munday et al., 2007).

Sadownik et al. (2012a, b), Buchan et al. (2007), and Brotto et al. (2013) all reported that their intervention groups, which consisted of multidisciplinary approaches including physiotherapy, psychotherapy, and mindfulness, contributed to an increased “sense of empowerment” (Sadownik et al., 2012a, p. 1091), “improved sense of self-efficacy” (Brotto et al., 2013, pp. 11–12), and “empowered them and gave them control of the condition” (Munday et al., 2007, p. 21), which in turn “served to lessen the emotional and psychological burden of their disease” (Brotto et al., 2013, p. 12). Further, Brotto et al. reported that following the intervention groups, “the theme of normalization was evident: women described a sense of relief in learning that they were not alone in their suffering and they directly credited this normalization to some of their subsequent improvements” (p. 12). This theme of normalization was also evident for pregnant women, who found that normalization around childbirth pain brought “some degree of comfort” (Johnson et al., 2015, p. 9–10).

#### Sub-concept 2: A Different Narrative

In two studies (Ayling & Ussher, 2008; Kaler, 2006), women who were able to adopt different narratives around womanhood, minimized experiences of “anxiety, fear, resentment, guilt, shame, isolation, and the positioning of both the body and self as ‘faulty’” (Ayling & Ussher, 2008, p. 301). The changes that were reported centred on adopting different and more helpful narratives around sex and finding new narratives of “how to be a woman.” Firstly, the one woman who was able to adopt “an egalitarian relational discourse,” which did not “privilege one partner’s needs or concerns over the others,” allowed her, and her partner, to “dismiss the ‘coital imperative,’ and experiment with other sexual practices,” which in turn freed this woman from the “physical and psychological pain” which had previously been linked with painful coitus (Ayling & Ussher, 2008, p. 299). However, it must be borne in mind that only one woman across the studies reported adopting a more helpful egalitarian discourse and reasons for this warrant further exploration. Secondly, some women rejected the notion that intercourse made them a woman, and instead adopted other (non-sexual) behaviors, particularly those associated with a traditional view of “womanhood,” such as mothering and caring, which enabled women to maintain the concept of “essential womanhood” (Kaler, 2006, p. 68). Similarly, Ayling and Ussher (2008) found that women attempted to renegotiate the construct of womanhood, instead emphasizing “caring qualities” and a “nurturing nature.”

Before the Discussion section, and in keeping with a meta-ethnographic methodology, the next section will explore the extent to which the papers were reciprocal and/or refutational. This reciprocal and refutational analysis helps inform the authors’ “third-order interpretations.” These “third-order interpretations” outlined in the “line of argument” section draw together the entirety of the analysis (including the reciprocal/refutational analysis) to produce new ideas, moving our understanding beyond the summation of the concepts from the original papers, contributing new insights into this topic area of vulvodynia.

### Reciprocal and Refutational Analysis

Overall, the studies do not refute each other. Despite this apparent lack of contradiction across the studies, papers which explicitly described and acknowledged the authors’ epistemological standpoint, utilizing methods of analysis in keeping with their epistemologies, tended to place the difficulties experienced by these women with vulvodynia within the context of (often unhelpful) cultural ideologies around sex, gender, and womanhood (Ayling & Ussher, 2008; Kaler, 2006; Marriott & Thompson, 2008). In contrast, papers using content/thematic analysis, with no reference to epistemological standpoint tended to place the difficulties within the individual, moving away from understanding within a cultural context and moving toward the individualized treatment of women from a variety of perspectives such as physiotherapy, medication, or local anesthetic products, psychotherapy, mainly in the form of CBT or mindfulness and dietary advice.

In this way, the studies comprised two groups, which (although sometimes over-lapping) are not in contradiction, but rather complement and mutually support each other:

- *“*Overt epistemology papers*”* primarily concerned with critical realist, feminist, and phenomenological perspectives (Ayling & Ussher, 2008; Kaler, 2006; Marriott & Thompson, 2008).
- *“*Thematic papers*”* primarily concerned with categorization and theming of qualitative data as a way of evaluating interventions (using a pre- and post-methodology), or experiences, not situated with a specified epistemological framework (Brotto et al., 2013; Buchan et al., 2007; Johnson et al., 2015; Munday et al., 2007; Sadownik et al., 2012a, b).

This may explain the different foci of conclusions each group of papers draws, with “overt epistemology papers” centering on the helpfulness of changes in narrative, while the “thematic papers” focus on individual experience, without taking into account social and cultural factors and the helpfulness of individualized multidisciplinary approaches. Interestingly, despite the differences, both changing narratives and individualized approaches allowed women to open up communication with others, which women resoundingly reported as being helpful and improving the feelings of shame, stigma, and associated low mood and anxiety.

### Line of Argument

Following this review, we can more firmly conclude that the women in the included studies experienced negative consequences of social narratives around womanhood, sexuality, and femininity, including the prioritization of penetrative sex, the belief that women’s role is to provide sex for men, and the portrayal of sex by the media as easy and natural. These discourses affected women’s relationships, both intimate heterosexual relationships and relationships with other women, from whom the women with vulvodynia felt excluded. Moreover, women experienced the healthcare system as dismissive, sometimes being prescribed treatments that exacerbated their pain. These experiences left women feeling silenced and isolated, and experiencing shame and guilt because of the social taboo, especially around female sexuality, which in turn led to the experience of psychological distress, low mood, anxiety, and low self-esteem.

However, the review also suggests that educating women about vulvodynia and empowering them to gain a sense of control over their experience of pain in the form of multidisciplinary group work (grounded within an individualistic approach) is helpful for women (Brotto et al., 2013; Buchan et al., 2007; Munday et al., 2007; Sadownik et al., 2012a, b), although the long-term outcomes of these groups are yet to be measured. Incorporating discussions around unhelpful social constructs may prevent individualized interventions (designed to “fix” women) unwittingly “blaming” or holding women solely responsible for the management of vulvodynia and its psychological consequences.

Overall, this review demonstrates that heteronormative patriarchal ideologies play a significant role in how the women in the included studies experience vulvodynia. Most importantly, it highlights the psychological impact of living with pain in an intimate area which is so inextricably tied up with sexuality and gender, in a culture that conflates sex with penis in vagina penetration and penis in vagina penetration with womanhood.

## Discussion

It is clear from reviewing the literature that women with vulvodynia described in numerous studies as subscribing to many unhelpful phenomena previously documented and explored by feminist sexology researchers since the 1980s (Jackson, 1984). In the majority of papers, women adhered to dominant notions of sexual acts focused on the coital imperative, phallocentrism, patriarchal ideologies, and the hydraulic male sex drive (Du Plessis, 2015; Exner et al., 2003; Gavey et al., 2001; Marken, 1996; McPhillips et al., 2001; Tiefer, 2001; Vitellone, 2000). In keeping with previous findings, women with vulvodynia are limited by these constructions as they leave little room for alternative ways of gaining sexual satisfaction and pleasure. Similarly to Du Plessis’ (2015) work regarding the media as an agent for dictating sexual normalcy, women seemed to be heavily influenced by media outlets when it came to assessing themselves in comparison to “the norm,” often reporting that it offered little in the way of alternatives to limited patriarchal narratives of sex. While simultaneously demonstrating that women are still hindered by patriarchal ideologies surrounding sex, the current review also demonstrates that a culminating consequence of such ideologies is that women with vulvodynia feel they are “not real women,” a theme that was described in the majority of studies. This same theme has been reported in previous research where women experience anorgasmia (Lavie & Willig, 2005), demonstrating the impact of being unable to subscribe to narrow patriarchal narratives of sexual normalcy.

Perhaps more worryingly, when interacting with the medical model, women with vulvodynia reported similar experiences to those previously documented in feminist sexology literature. For example, women felt they were patronized by medical professionals because of their gender, which is in keeping with previous literature detailing the paternalistic attitudes of doctors toward women (Gannon, 1998). Moreover, the current study also supports previous quantitative research that demonstrates the lack of knowledge among doctors surrounding vulvodynia (Toeima & Nieto, 2011) with the majority of papers reporting this experience by women. Consequently, understanding doctors’ lack of awareness of, and negating attitude toward vulvodynia, as impacting directly upon increased distress and worsening experience of vulvodynia, is something this review adds. This notion that interacting with healthcare services may be causing more harm than good for women warrants further, closer examination.

The qualitative literature reviewed here supports and is supported by previous quantitative literature pertaining to the psychological consequences of living with vulvodynia (Gates & Galask, 2001). However, by examining previous feminist sexology literature and qualitative literature on women’s experiences of vulvodynia, we begin to understand the complexity of these psychological difficulties, with patriarchal ideologies, media portrayals of sexual normalcy, and interactions with uninformed and negating medical professionals all likely to be contributing to the psychological consequences, perhaps above and beyond the experience of pain itself (Marriott & Thompson, 2008). Further, the media promotes innate, inflexible accounts of male sexuality as being in need of penetration and prioritization, thus silencing any communication on alternative ways of having sex, preventing the exploration of pleasure for both women with vulvodynia and their partners.

### Implications

The findings from the current review demonstrate that the difficulties women with vulvodynia face need to be addressed twofold: firstly by hypothesizing what treatments may be helpful for individual women, especially relating to the psychological impact of living with vulvodynia; and secondly, and perhaps most importantly, by proposing where changes in unhelpful social construction and narratives could be targeted.

The current review highlights shame as a particularly predominant psychological difficulty that arose for many of the women within the reviewed literature. Thus, it could be postulated that therapeutic models of shame (see Gilbert, 2006) may be particularly helpful in understanding the psychological experiences of women with vulvodynia, which conceptualize shame as “the underlying desire to be valued and seen as a talented, deserving, desirable individual” (Gilbert, 2006, p. 87). Formulating vulvodynia using models of shame, and intervening, using for instance, compassion-based therapy (Gilbert, 2006), may be one helpful way to begin to alleviate psychological difficulties in women with vulvodynia. Similarly, the importance of changing narratives was highlighted across the studies as a particularly helpful action for women with vulvodynia. Therefore, it could also be tentatively concluded from this review that therapies from a constructionist perspective such as solution-focused therapy (De Shazer & Dolan, 2007), cognitive behavioral therapies (Hawton, Salkovskis, Kirk, & Clark, 1989), and narrative therapy (White & Epston, 1990) may also be useful for women with vulvodynia, helping them recognize and challenge unhelpful narratives developed within a cultural context (Harper & Spellman, 2006). Finally, given the relational nature of the distress associated with vulvodynia, couples therapy may also be of benefit and, along with the other therapies mentioned, is worthy of future research to ascertain effectiveness within this client group.

However, given the recommendations drawn from the findings of this review, interventions should not solely be aimed at the individual level (i.e., challenging women’s negative—implied faulty—thinking, or working on their pelvic floor muscles or learning how to have pain-free intercourse), based on the notion of “fixing” women, but rather using psychological perspectives to help women (their partners, healthcare professionals and any further important others) understand and begin to deconstruct unhelpful narratives and reconstruct more helpful ones around vulvodynia, gender, and sexuality that begin to challenge ingrained societal scripts. In keeping with this, Du Plessis (2015) concludes that both media and primary healthcare professionals should reintroduce sex within as wide a definition as possible that affirms pleasure over the whole body and encourage people to explore and experiment with pleasurable feelings associated with non-genital erogenous zones in order to disestablish the dominance of phallocentric versions of sex. In doing so, there is the potential to empower both men and women to create mutual sexual relationships based on communication and respect (Du Plessis, 2015).

Similarly, the World Health Organization’s sexual health programs recognizes that the media, primary healthcare professionals, education systems and societies as a whole, all have a role to play in providing information that is “free from discrimination, gender bias and stigma” (WHO, 2010). Specifically, some particularly pertinent suggestions for action include training health professionals and teachers in sexuality and sexual health, promoting interventions that influence social norms and promote gender equality, particularly through media (radio & TV programs) and providing health services that are comprehensive (WHO, 2010). The authors from this review would add that training for health professionals regarding sexuality and sexual health should also include training around patriarchal ideologies and that providing comprehensive health services should also encompass a knowledge of vulvodynia at the primary care level, but also the provision of specialist vulvodynia services at secondary and tertiary levels that are easily accessed.

### Methodological Considerations

There are several limitations to the current research. Although systematic review guidance was utilized in order to ensure the rigorous searching procedures outlined above, it is possible that not all relevant literature were identified. The review only included studies published in English and in peer-reviewed journals, excluding any research in other languages or research in other sources, such as theses/dissertations, the inclusion of which would have been beyond the scope of the current review. The current paper excluded research whereby women had been interviewed with their partners, for several reasons: first of all, women interviewed in the presence of a partner may feel unable to give true and accurate accounts of their experiences, either consciously or unconsciously, and secondly, the experience of partners was not the focus of this review. For these reasons, the experience of couples would be better served by a separate review. The studies reviewed only offer insights gained from predominantly young (i.e., pre-menopausal), white, educated, heterosexual women living in Westernized, English-speaking societies, who have access to health services. Therefore, the findings and conclusions of this review cannot be assumed to apply to other groups of women differing in culture, education, socioeconomic status, ethnicity, and healthcare access.

Despite these limitations, the current review has several strengths, including the utilization of a strict and rigorous systematic searching in line with PRISMA guidance (Moher, Liberati, Totzlaff, Altman, & The PRISM Group, 2009). The Critical Appraisal Skills Programme (2014) tool, a well-recognized and widely used quality assessment tool, was utilized in order to quality assess papers to provide context for the included papers. Finally, in-depth analysis and synthesis of the data was undertaken, rather than simple categorization of the findings, allowing the current review to contribute new insights, furthering our understanding of vulvodynia, and allowing reliable and useful implications for clinical practice to be drawn.

### Future Research

Following this review, several areas warrant further investigation in order to begin to improve this under-researched and under-recognized health condition. This includes research with women from different ethnic and cultural backgrounds, asexual, bisexual and lesbian women and women who are postmenopausal. Moreover, the journey toward diagnosis was mentioned consistently throughout the papers as being particularly problematic for women with vulvodynia, impacting upon psychological well-being. There is only one paper focused on this specific topic. Unfortunately, due to methodological weaknesses, it is difficult to draw conclusions about how and why this journey is particularly problematic and distressing for women. Therefore, further research into women’s experiences of the journey toward diagnosis and its impact upon the psychological well-being of women is warranted, so that reliable and firm conclusions can be drawn in order to improve this process for women with vulvodynia. Research into devising, implementing, and evaluating psychological interventions, based on the observations made in the current review, is warranted. It would be helpful to review existing research into the effectiveness of psychological interventions for women with vulvodynia as a starting point for developing both one-to-one therapy and group therapy for secondary psychological difficulties suffered by these women. Finally, research focused on changing and broadening notions of sexuality within healthcare, education and media institutions and how this may benefit participants with vulvodynia is of the utmost importance.

### Conclusion

Qualitative research pertaining to the experiences of women with vulvodynia consistently reports psychological difficulties. From reviewing the literature, it seems that interventions aimed at helping the individual woman’s experience of pain, as well as interventions aimed at changing social narratives, may be helpful for the psychological well-being of women with vulvodynia.
